# Variation of Vitamin D in Cow’s Milk and Interaction with β-Lactoglobulin

**DOI:** 10.3390/molecules180910122

**Published:** 2013-08-22

**Authors:** Omar Bulgari, Anna Maria Caroli, Stefania Chessa, Rita Rizzi, Carmen Gigliotti

**Affiliations:** 1 Department of Molecular and Translational Medicine, University of Brescia, Brescia 25123, Italy; E-Mails: bulgari.omar@libero.it (O.B.); giglio@med.unibs.it (C.G.); 2 Institute of Agricultural Biology and Biotechnology—National Research Council, Milano 20133, Italy; E-Mail: chessa@ibba.cnr.it; 3 Department of Veterinary Science and Public Health (DIVET), Milano 20133, Italy; E-Mail: rita.rizzi@unimi.it

**Keywords:** vitamin D, bovine milk, β-lactoglobulin

## Abstract

Vitamin D is the collective name for a group of closely related lipids, whose main biological function is to maintain serum calcium and phosphorus concentrations within the normal range by enhancing the efficiency of the small intestine to absorb these minerals from the diet. We used a commercially available ELISA method for the determination of vitamin D in bovine milk. Individual milk samples from two different Italian Friesian herds were analysed. The enzyme immunoassay method used was confirmed as a useful tool to measure the vitamin D in the milk as it greatly reduces the time required to perform the conventional HPLC analysis. An interesting variation was found among individual animals that may be associated with management factors and specific genetic effects. A relationship was highlighted between vitamin D and the genetic polymorphism of β-lactoglobulin, the main bovine whey protein which is involved in the transport of small hydrophobic molecules such as retinol and vitamin D. The relatively high content of vitamin D in most milk samples suggests an opportunity to improve the natural content of vitamin D in milk either by acting on the herd management or selecting individuals genetically predisposed to produce milk with a higher vitamin D content.

## 1. Introduction

Vitamin D is the collective name for a group of lipids which are closely related. The two major forms are vitamin D_3_, or cholecalciferol, synthesized by the skin of vertebrates following exposure to ultraviolet radiation (UV) B, and the vitamin D_2_ or ergocalciferol, produced in various plants, yeasts and fungi, always due to exposure to UV B radiation [1].

In the presence of sufficient light, the vitamin D_3_ is formed in a non-enzymatic way in the skin from 7-dehydrocholesterol. Vitamin D_2_ has an additional methyl group with respect to the D_3_ and is formed from ergosterol, following a biochemical process similar to that of vitamin D_3_ [1]. The active form of vitamin D_3_, the 1,25-dihydroxycholecalciferol [1,25(OH)_2_D_3_] or calcitriol, is formed from vitamin D_3_ through two independent hydroxylation reactions, in the liver and in the kidneys; vitamin D_2_ is activated in a similar way.

In temperate latitudes, sun exposure can guarantee 80% of the human requirement of vitamin D, while the remaining 20% is obtained through the diet [2]. Vitamin D_3_ is contained almost exclusively in animal fats, while the proportion of vitamin D_2_ present in some vegetable fats is negligible. Vitamin D introduced with food is rapidly absorbed in the duodenum and jejunum and, subsequently, distributed, through the lymphatic circulation, almost completely to the adipose tissue, from which is released in small quantities compared to the stored portion [3].

The importance of an adequate intake of vitamin D for bone health is well known [4]. The main function of the active vitamin D is to maintain the concentrations of calcium and phosphorus in the blood within a normal range of variability by improving the efficiency of the small intestine to absorb these minerals from the diet [5,6].

In the kidney, 1,25(OH)_2_D increases the resorption of calcium from the distal renal tubules. In addition to these consolidated actions associated with the metabolism of calcium, 1,25(OH)_2_D and similar synthetic compounds are increasingly recognized for their potent anti-proliferative, pro-differentiation and immuno-modulatory action [7]. In addition, vitamin D plays important roles in promoting oral health and preventing colon cancer [8]. In recent years increasing attention has been paid to its requirements, in order to optimise important aspects of health such as the neuromuscular and immune function [1,9].

The Fourteenth Vitamin D Workshop (Brugge, Belgium) consensus on vitamin D nutritional guidelines established that an absolute minimum 25(OH)D level of 20 ng/mL is necessary in all individuals to support and maintain all of the classic actions of vitamin D on bone and mineral health. Less consensus exists on the proposal that plasma levels <30 ng/mL would indicate a vitamin D deficiency [10].

As already highlighted, the main portion of vitamin D_3_ derives from the conversion of 7-dehydrocholesterol upon exposure to ultraviolet rays. Several factors may reduce the exposure of an individual to sunlight [10,11]. Therefore, vitamin D derived from the diet and/or specific supplements plays a crucial role in obtaining optimal physiological levels [12]. The recommended dietary allowance (RDA) for vitamin D is 600 IU/day for individuals aged between 1 and 70 years, and 800 IU/day for ages above 70 years [13].

Vitamin D naturally present in the diet in larger quantities is cholecalciferol (vitamin D_3_) contained in foods such as oily fish [14]. Lower quantities of vitamin D_3_ and its metabolite, 25-hydroxyvitamin D_3_, are found in meat, eggs [15,16] and other foods of animal origin, including milk and dairy products. In many countries milk is fortified, mainly by adding vitamin D_3_. Most of the milk sold at retail in the United States is fortified, while the fortification is less common in Europe. The final concentration of vitamin D_3_ in fortified milk in the United States is 400 IU (10 µg) per quart (qt, 1 qt = 946.4 mL) [17]. Because of its high consumption, the fortified milk is the major dietary source of vitamin D [14,18].

The aim of this paper was to quantify the vitamin D_3_ naturally occurring in individual milk samples with a commercial ELISA kit, analysing the main causes of variability of its content. The special attention given to individual milk arises from the interest to identify animals genetically predisposed to produce milk naturally associated with higher levels of vitamin D_3_. Moreover, some commercial milk samples were analysed as a reference test for the use of the kit.

## 2. Results and Discussion

### 2.1. Content of Vitamin D in Commercial Milk Samples

The following commercial milk samples were analysed: A raw milk sample, two unfortified Ultra High Temperature (UHT) milk samples, and one fortified UHT milk sample. The raw milk sample contained 0.57 IU/mL of vitamin D_3_, a higher value, as expected, than the activity observed in two samples of unfortified UHT milk, which contained respectively 0.34 IU/mL and 0.44 IU/mL of vitamin D. The content of vitamin D in the fortified milk was equal to 0.80 IU/mL, a value slightly greater than the upper limit of sensitivity (0.75 IU/mL) of the method.

The difference between the vitamin D in the fortified UHT sample (0.80 IU/mL) and the average of the two unfortified UHT milks (0.39 IU/mL) was equal to 0.41 IU/mL. Since 40 IU of vitamin D correspond to 1 μg of the compound, the difference found corresponds to 0.011 µg/mL of vitamin D and is comparable to the quantity of vitamin D added to milk and declared on the packaging, equal to 10 µg/L. The enzyme immunoassay method used was confirmed as a useful tool to measure the vitamin D in the milk as it greatly reduces the time required to perform the conventional HPLC analysis.

### 2.2. Content of Vitamin D_3_ in Individual Milk Samples: Differences between Herds

**Figure 1:**
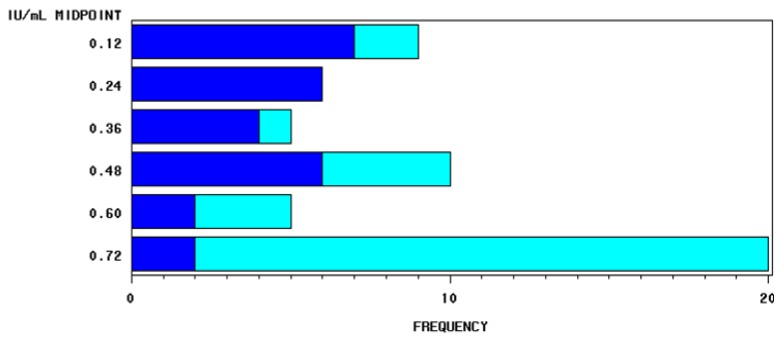
Frequency distribution of the activity of vitamin D_3_ in 55 individual milk samples from Italian Friesian cows as a function of the two different herds (blue and cyan).

An interesting variation was found among individuals that may be associated with management factors as well as physiological and genetic effects. The difference in the distribution of the milk activity of vitamin D_3_ between the two sampled herds is shown in Figure 1.

The difference between herds (herd B *vs.* herd A: + 0.29 IU/mL corresponding to + 0.007 μg/mL of vitamin D_3_) was significantly different (*p* < 0.0001). Table 1 shows the descriptive statistics of the quantification of vitamin D in the Italian Friesian individual milk samples analysed. Approximately 12.7% of the samples showed values of vitamin D_3_ less than or equal to 0.125 IU/mL, while the 32.7% of the samples showed values greater than or equal to 0.75 IU/mL. In the first case, the observations were concentrated in herd A, while in the second they were concentrated in herd B.

This difference may be due to managerial factors such as exposure to the sun and feeding the cows. Reeve and coauthors [19] analyzed the different forms of vitamin D in milk from cows that were administered different doses of vitamin D daily: 15,000 IU, IU 65,000, 115,000 and 215,000 IU. A higher nutritional intake of vitamin D corresponded to an increase of vitamin D in the milk, however, the authors observed that a 14-fold increase of vitamin D in the diet only corresponded to a two-fold increase in the activity of vitamin D in the milk.

**Table 1:** Descriptive statistics of the content of vitamin D_3_ in milk individual samples of the 55 Italian Friesian cows.

Vitamin D_3_	Herd A		Herd B		Herd A + B	
	n = 27		n = 28		n = 55	
Measure unit	IU/mL	µg/mL	IU/mL	µg/mL	IU/mL	µg/mL
Mean	0 *.*341	0 *.*008	0 *.*629	0 *.*016	0.487	0.012
Standard Deviation	0.192	0.005	0.184	0.005	0.237	0.006
Median	0 *.*311	0 *.*008	0 *.*750	0 *.*019	0.505	0.013
Minimum value	0.125	0.0031	0.125	0.0031	0.125	0.0031
Maximum value	0.750	0.0187	0.750	0.0187	0.750	0.0187
Samples ≤ 0.125 IU/mL	10.91%		1.82%		12.73%	
Samples within the range of sensibility	34.55%		20.00%		54.55%	
Samples ≥ 0.75 IU/mL	3.64%		29.09%		32.73%	

For lactating cows, dietary intake of vitamin D equal to 15,000 IU/day, which was considered adequate in 1978 [20], but was later revised [21]. In fact, the definition of the appropriate dietary intake of vitamin D is difficult to define, since animals exposed to sunlight, at certain latitudes, rather than animals fed with hay exposed to the sun, may not require dietary supplements of this vitamin [21]. Horst and coauthors [22] defined a plasma concentration of 25-hydroxyvitamin D of less than 5 ng/mL as indicative of a deficiency of vitamin D, while levels of 25 (OH) D between 200 and 300 ng/mL indicative of toxicity. Normal cows have concentrations of 25-hydroxyvitamin D in plasma between 20 and 50 ng/mL [22]. In most cases, a daily intake of 10,000 IU (16 IU vitamin D/kg live weight) should provide an adequate level of vitamin D in cows in late lactation. On the basis of all available data, the requirement of 30 IU/kg body weight was established in 1989 [23] seems justified [21]. This requirement is approximately equivalent to a daily intake of vitamin D equal to 18,750 IU.

In cattle, hypovitaminosis D, reducing the ability to maintain homeostasis of calcium and phosphorus, has resulted in a decline in the blood level of phosphorus and, less frequently, a decrease in the level of plasma calcium. Hypovitaminosis D is therefore eventually associated with rickets in young animals and osteomalacia in adults [21]. Conversely, vitamin D intoxication is associated with a reduced food intake, to polyuria followed by anuria, to dehydration of the faeces and reduced milk production. At necropsy calcification occurs at the level of kidneys, aorta, abomasum, and bronchioles [24].

### 2.3. Content of Vitamin D_3_ in Individual Milk Samples: Physiological and Genetic Differences

Among the other sources of variability considered, the level of somatic cells of the milk expressed as Somatic Cell Score (SCS) and the genotype of β-lactoglobulin presented a statistically significant effect on the vitamin D content. The SCS is a logarithmic transformation of the somatic cell count (SCC) of milk per mL [SCS = log base 2 (SCC/100,000) + 3] which aims to normalize the distribution of SCC [25].

With an increase of SCS, the content in vitamin D decreases in a statistically significant way (*p* < 0.05), with a linear regression coefficient equal to −0.027 IU/mL of vitamin D_3_ per point of SCS. Since milk somatic cells are closely associated with the state of health of the mammary gland [26], the observed effect can be explained by the fact that a higher content of vitamin D is presumably associated with a better state of health of the animal and, in particular, of the mammary gland.

No significant effect was found for lactation number and days in milk. An interesting relationship was highlighted between vitamin D_3_ content and the genetic polymorphism of β-lactoglobulin (β-LG), the main whey protein of cow's milk which is involved in the transport of small hydrophobic molecules such as retinol and vitamin D [27]. The AA genotype was associated to a content of vitamin D_3_ significantly higher (*p* < 0.01) than the pool of the other two genotypes, the heterozygous AB and the homozygous BB (Table 2).

**Table 2:** Effects of β-lactoglobulin (β-LG) genotype on the content of vitamin D_3_ in bovine individual milk samples. Least-square means (LSM) with different letters are significantly different (p < 0.01).

β-LG genotype	Measure unit	Number	LSM	Standard error
AA	μg/mL	19	0.015 ^a^	0.001
AB + BB	μg/mL	36	0.011 ^b^	0.001
AA	IU/mL	19	0.602 ^a^	0.051
AB + BB	IU/mL	36	0.427 ^b^	0.037

**Figure 2:**
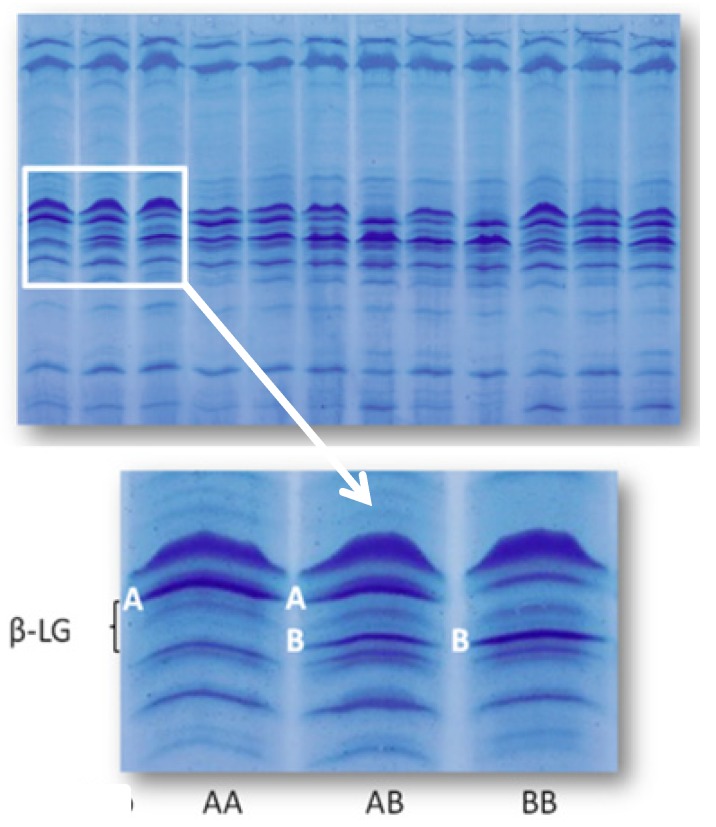
Isoelectrofocusing of individual milk samples from Italian Friesian cows. A particular showing the three most common bovine β-lactoglobulin (β-LG) genotypes is highlighted, showing the higher expression of β-LG A variant.

This result is not surprising. The two main genetic variants of bovine β-lactoglobulin are A and B, differing for two amino acid substitutions only. The higher expression of variant A is well known [28]. In several studies, starting from Cerbulis and Farrell [29], a greater protein expression level of the β-LG A variant compared with the B variant has been reported. The functional reason for differences in expression between the two alleles has been investigated by several authors who highlighted significant differences within the non coding region of the gene coding for β-lactoglobulin [28]. Figure 2 shows the different expression associated with the two variants of β-lactoglobulin and the resulting genotypes.

The ability of β-LG, a protein that belongs to the lipocalin family, to bind the vitamin D has long since been demonstrated [30]. Forrest and coauthors [31] have further characterized the interaction between vitamin D_3_ and milk proteins choosing, among these, the β-LG A and β-casein which represent, respectively, the most abundant whey protein and casein. Both proteins bind strongly to vitamin D; this fact can greatly affect the stability and therefore the bioavailability of this vitamin in dairy products. The same authors suggest that vitamin D_3_ can bind to β-lactoglobulin A in the production of fermented milk-based products (cheese, yoghurt) or in the acid environment of the stomach. The greater amount of β-LG expressed by the A variant [28] can justify the higher content of vitamin D_3_ associated with the homozygous AA genotype.

The relatively high content of vitamin D_3_ in the samples analysed justifies the limited use of the fortification of milk in Italy and suggests an opportunity to improve the content of vitamin D naturally present in milk, either by acting on the herd management or evaluating the possibility to select individuals predisposed to produce milk with a higher vitamin D content.

## 3. Experimental

A total of 55 individual samples of unfortified cow’s milk from two different herds were analysed as well as different types of milk for human consumption available on the market: A sample of raw milk, two samples of UTH milk, and a sample of fortified UHT milk (the content of vitamin D stated on the label was equal to 10 µg/L).

In collaboration with the Provincial Breeders Association (APA) of Brescia, 55 individual milk samples were collected from Italian Friesian cows belonging to two different farms in the province of Brescia (northern Italy). Milk samples were taken in the second half of the month of May, 2012, during the morning milking and immediately frozen at −20 °C for subsequent analyses.

**Figure 3:**
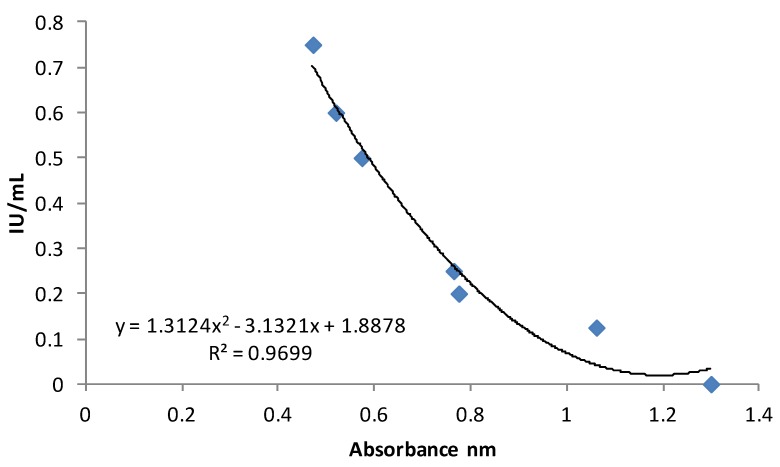
Calibration curve of the vitamin D_3_ content (IU/mL) in milk referred to the absorbance values obtained by the commercial ELISA kit.

The samples were analysed with the VitaKit DTM (SciMed Technologies Inc., Edmonton, Alberta, Canada). The Life DTM Kit is a competitive enzyme immunoassay ELISA developed for the determination of vitamin D_3_ after extraction. The assay uses a known amount of vitamin D immobilized in solid phase present in the ELISA plate. Vitamin D acts as a competitive substrate with the unknown amount of vitamin D_3_ extracted from milk samples of a fixed number of sites of the labeled monoclonal antibodies that recognise vitamin D_3_ specifically. The absorbance was measured by a microplate reader (Model 680, Bio-Rad Lab Inc., Hercules, CA, USA) at 450 nm. A calibration curve was obtained in order to relate the absorbance of the standard samples provided in the kit with the respective known concentrations of vitamin D_3_ (Figure 3).

The high determination coefficient obtained (R^2^ > 0.96) indicates the good fitness of the prevision equation to the data. Such equation was used to quantify vitamin D_3_ in the milk samples successively analysed. On the basis of the indications provided by the kit, the sensibility of the method ranges from 0.125 IU/mL to 0.75 IU/mL. The inter-assay and the intra-assay coefficient of variation are 8% and 4%, respectively.

The 55 individual milk samples were also analysed to genotype milk protein loci, including β-LG, by isoelectrofocusing [32]. The numbers of cows per genotype were 19 for β-LG AA (herd A = 5, herd B = 14), 22 for β-LG AB (herd A = 13; herd B = 9), and 14 for β-LG BB (herd A = 9; herd B = 5).

Statistical analysis was performed by General Least-Square Mean (GLM) procedure of SAS (SAS Institute Inc, 2008, SAS OnlineDoc^®^ 9.1.3, Cary, NC, USA) to evaluate the effect of the following variables on the content of vitamin D: Herd, lactation number (range = 2 to 5), days in milk (range = 100 to 269), milk somatic cell score (range = 0.06 to 8.83), as well as the genotype at β-lactoglobulin locus.

## 4. Conclusions

First, from a methodological point of view, the enzyme immunoassay used is a useful tool to measure vitamin D_3_ in milk by greatly reducing the time required to perform the conventional analysis by HPLC. Secondly, an interesting variation was found between individuals. The two herds analysed showed a considerable variability that may be associated with factors to corporate management, as well as to specific genetic effects. In this respect, interesting was the relationship emerged between vitamin D and genetic polymorphism of β-lactoglobulin, which was also found to be unbalanced in the two herds. Other candidate genes as well as Single Nucleotide Polymorphisms (SNP) along the bovine genome should be investigated within a wider study in order to highlight the significant effects of their variability on the amount of vitamin D naturally present in milk for human consumption.
